# Clinical Conditions of Hospitalized Older Adult Patients and Their Outcomes in a Regional Referral Hospital in Southwestern Uganda

**DOI:** 10.1155/2020/6830495

**Published:** 2020-06-29

**Authors:** Patrick Orikiriza, Godfrey Z. Rukundo, Adrian Kayanja, Joel Bazira

**Affiliations:** ^1^Department of Microbiology, Mbarara University of Science and Technology, Mbarara, Uganda; ^2^Division of Basic Medical Sciences, University of Global Health Equity, Kigali, Rwanda; ^3^Department of Psychiatry, Mbarara University of Science and Technology, Mbarara, Uganda; ^4^Department of Internal Medicine, Mbarara Regional Referral Hospital, Mbarara, Uganda

## Abstract

**Background:**

Recent advances in medicine have caused positive impact on the life expectancy of most countries, resulting in increased older adult population. Aging comes with a number of health challenges. This study investigated health conditions of older adults at admission and clinical outcomes in a regional referral hospital in southwestern Uganda.

**Methods:**

A retrospective study reviewed clinical data of older adult patients admitted between January 2016 and December 2017. Demographic data, cause of admission, length, and outcomes of hospitalization are described.

**Results:**

Up to 813 patient files were reviewed. The patients had been hospitalized to emergency, 371 (45.6%); medical, 355 (43.7%); surgical, 84 (10.3%); psychiatry, 2 (0.3%); and obstetrics and gynecology, 1 (0.1%) wards. The majority, 427 (52.5%), of the patients were females. Cancer was the most common reason for hospitalization, 130/889 (14.6%), followed by stroke, 94/889 (10.6%); heart failure, 76/889 (8.6%); chronic obstructive pulmonary disease, 56/889 (6.3%); pneumonia, 47/889 (5.3%); and head injury, 45/889 (5.1%), whilst 560 (68.9%) of the hospitalized patients were discharged, 197 (24.2%) died, 18 (2.2%) were referred for advanced care, and 38 (4.7%) escaped from the facility. The emergency ward had the highest deaths, 101 (51.3%), then medical, 56 (28.4%), and surgical, 39 (19.8%), wards. Mortality of those who died was admitted with stroke, 30 (15.2%), cancer, 21 (10.7%), head injury, 16 (8.1%), heart failure, 14 (7.1%), sepsis, 14 (7.1%), and renal disease, 12 (6.1%). On average, patients were admitted for 5 days (IQR: 3–8).

**Conclusions:**

The high proportion of mortality in this group is worrying and requires further investigations.

## 1. Background

Globally, aging populations are emerging at a rapid rate due to the general increase in life expectancy following advance in medicine and improved health systems in most countries [1]. The United Nations Definition of Older Persons includes individuals aged 60 years and more [2].

In the 2016 WHO report, the global life expectancy was estimated at 72 years: 74.2 years for females and 69.8 years for males [3]. An annual growth of 3% has been noted among the older adult population [4], and many countries are expected to witness a similar trend over the years. In line with this, the global population of older adult people in 2017 was estimated to be 962 million people and projected to reach nearly 2.1 billion by 2050 [5], and the majority of whom (two-thirds) are in developing countries.

Old age comes with a number of medical and social challenges that need appropriate and timely interventions [5] to allow active participation in societal development. Indeed, studies have shown that older adult people contribute to up to 23% of the total global burden of diseases [6]. Also, more evidence particularly from developed countries indicate that older adult people are hospitalized more frequently and for a longer period of time than any other age groups as they tend to suffer more severe forms of diseases that are difficult to diagnose and treat [7, 8]. This can be highly challenging in low resource settings as it continuously impacts on their psychological, social, economic abilities and that of the health system [9].

Even in developed countries where health services are much improved, challenges among older adult people are common. In one study, it has been reported that one-third of hospitalized older adult patients face more complication by the time of discharged, including reduced functional capacity, than at admission [10]. The study further reveals that approximately 5% of these die during hospital stay and 20%–30% pass away within one year after hospital discharge. This indicates that there is a much bigger hidden problem that needs to be urgently addressed in order to meet the WHO call for the Universal Health Coverage.

Although the WHO attributes most of the health problems of the older adults to chronic diseases [11], very few studies have investigated the specific type of conditions and extent of this burden in low resource countries. To properly address these concerns, it is important to study the common conditions so as to establish specific interventions.

Uganda is already experiencing an increase in the population of older adult people as a result of improved life expectancy to 62.5 years [12]. Indeed, in the 2017 WHO report, the older adult people constituted approximately 1425000 (3.3%) of general population and this number is expected to quadruple by 2050 [5]. Despite this increasing trend, most health facilities lack dedicated services for this particular population. Indeed, there is limited evidence on health conditions of hospitalized older adult patients in Uganda.

With accurate data on the profiles of older adult patients and the common diseases that cause their hospital admissions, policies can be enacted to adequately provide necessary human and infrastructural resources required to meet health needs of this age-group as emphasized in the holistic approach of the global agenda for sustainable development [5].

This study is among the fast to highlight the health plight of hospitalized older adult patients in a rural hospital in Uganda. The study also described the outcomes and common medical conditions that resulted in mortality among these patients.

## 2. Methods

We reviewed all medical records of older adult patients admitted between January 2016 and December 2018, on medical, psychiatry, obstetrics and gynecology, surgical, and emergency wards within Mbarara Regional Referral Hospital (MRRH) in southwestern Uganda. Demographic data, proportion of older adults, health reason, duration, and outcomes of hospitalization were described from the records. This was an exploratory study, and therefore sample size was not estimated. Instead, all the records within two years were judged to give reasonable evidence. As a quality control, each file was reviewed by two study research assistants and verified by the principal investigator.

Patient records were maintained at the strictest level of confidentiality by all members of the study team. On all data collection documents, patient identifiers such as names were concealed and replaced with unique study identification numbers and these records were maintained in locked file cabinets.

The study was permitted by the MRRH administration and approved by the Research Ethics Committee of the Mbarara University of Science and Technology (18/08-18) as well as the Uganda National Council for Science and Technology (HS323ES).

## 3. Statistical Analysis

A case report form comprising of patient demographic data and clinical conditions was double entered into EpiData and analyzed using STATA version 13 (Texas, USA). Descriptive statistics were expressed as proportions in form of tables and graphs.

## 4. Results

We reviewed a total of 813 files during the study period between September and March 2019. Among those with gender records, 427 (52.5%) were females and the median age was 70 (IQR: 65–80). As shown in Table 1, most of the patients were married, 352 (43.3%), with no formal education, 283 (34.8%), and admitted by self-referral, 634 (78%). The majority of patients were admitted to the emergency ward, 371 (45.6%), and medical ward, 355 (43.7%).

The most common reasons for hospitalization were as follows: cancer, 130 (14.5%), stroke, 94 (10.5%), heart failure, 76 (8.5%), chronic obstructive pulmonary disease, 56 (6.2%), pneumonia, 47 (5.2%), head injuries, 45 (5.0%), diabetes, 43 (4.8%), hypertension, 36 (4.0%), sepsis, 35 (3.9%), severe anemia, 31 (3.4%), and severe malaria, 25 (2.8%), as shown in Table 2.

While 560 (68.9%) of the hospitalized patients were discharged, 197 (24.2%) died, 18 (2.2%) were referred for advanced care, and a large proportion, 38 (4.7%), escaped from the facility (Table 3).

The emergency ward had the highest deaths, 101 (51.3%), followed by medical, 56 (28.4%), and surgical, 39 (19.8%), wards (Table 4).

Most of the patients died from stroke, 30 (15.2%), cancer, 21 (10.7%), head injury, 16 (8.1%), heart failure, 14 (7.1%), sepsis, 14 (7.1%), and renal disease, 12 (6.1%) (Table 5).

**Figure 1:**
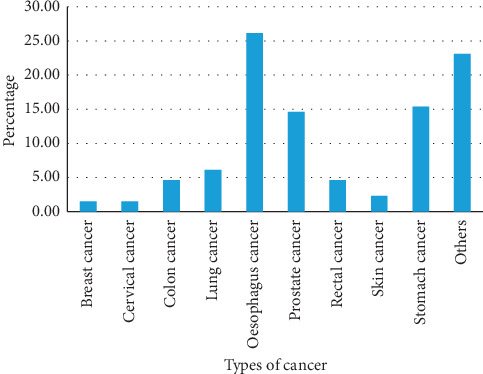
Common types of cancers observed at admissions at Mbarara Regional Referral Hospital.

As shown in Figure 1, cancer of the oesophagus was the most common form of cancer at admission, 34/130 (26.2%), followed by stomach cancer, 20/130 (15.4%), and prostate cancer, 19/130 (14.6%).

**Figure 2:**
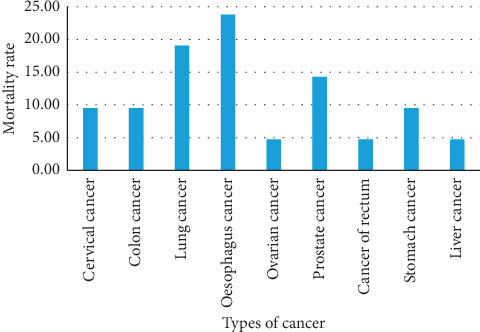
Cancers associated with mortality among older adult population at Mbarara Regional Referral Hospital.

Most of the people who died from cancer were diagnosed at admission with the majority presenting with esophageal cancer, 5/21 (23.8%), lung cancer, 4/21 (19.1%), prostate cancer, 3/21 (14.3%), cervical cancer, 2/21 (9.5%), colon cancer, 2/21 (9.5%), stomach cancer, 2/21 (9.5%), and rectal, ovary, and liver cancer, 1/21 (4.8%) each, in that order (Figure 2).

Finally, an average time of 5 days (IQR: 3–8) of admission was observed among this population.

## 5. Discussion

This study investigated the health conditions that caused older adult people to be admitted at a regional hospital in a semiurban community of southwestern Uganda. The study highlights critical issues and eventual outcomes. According to our knowledge, this is the first study to bring attention to the heath plight of this particular population.

First, we have shown that close to 1000 older adult people were hospitalized in this regional hospital within two years. The male to female ratio was also close to 1, which is similar to what we observed in our previous tuberculosis study among adults aged 18 and above, in the same setting [13].

Secondly, our study has shown that cancer, stroke, heart failure, chronic obstructive disease, pneumonia, and head injury dominated as most common conditions causing hospitalization. The raise in the number of cancer cases in the country has been previously described in the general population [14]. The rates reported in this study are in tandem with national figures [12]. Other countries have reported a different disease spectrum in a similar population. In one of the few African studies, hypertension, heart failure, ischemic heart disease, and anemia were reported as the most predominant causes of hospitalization [15]. Besides diabetes, the main cause for hospitalization in Europe, the other main conditions include cardiovascular diseases and pulmonary diseases, which are similar to what we observed in this study [16]. Indeed, by grouping the health conditions observed, cardiovascular diseases such as stroke, heart failure, and hypertension together contributed up to 29.2% of disease burden. Another study reported these cardiovascular diseases (30.3%) as the leading contributors to disease burden in older adult people followed by cancer (15.1%) and chronic respiratory diseases (9·5%) [6]. Although different context, the proportions are much similar, indicating that addressing these health challenges could greatly minimize overall risks of hospitalization in many settings.

In the outcome analysis, this study observed an in-hospital mortality rate of 24.2% while 68.9% were discharged. Our findings on mortality is much higher than 8.5%, 12%, 16.4%, and 14.9% previously reported in Turkey, Portugal, Brazil, and Italy [16–19] in a similar population but close to what was observed in Nigeria (18.7%) within a related teaching hospital [20]. Again, our findings are the first to show such a high mortality in this population and require deeper investigations in future studies.

We further investigated the most common health conditions that were observed among the patients that died during hospitalization. Stroke (15.2%), cancer (10.7%), head injury (8.1%), heart failure (7.1%), sepsis (7.1%), and renal disease (6.1%) accounted for the highest incidences. Indeed, the WHO predicts that by 2020, noncommunicable diseases such as heart disease, cancer, and diabetes will be among the main causes of mortality in the African region [21]. Already high incidences of cerebrovascular accident (25.1%) have been reported predominantly besides malignancies (15.2%), diabetes mellitus (8%), and congestive cardiac failure (6.2%) [20]. Others include septicemia (5.2%), trauma (4.6%), renal failure and chronic obstructive pulmonary disease (3.9%).

Currently, a joint extract from the WHO, World Bank, and UNESCO, reports HIV/AIDS (13.3%), pneumonia (11%), diarrheal diseases (6.4%), malaria (4.8%), and stroke (4.7%) among the most common causes of death in the general population in Uganda [12]. This is much different from what we observe in this study indicating that the two populations are completely unique. In addition, this adds more evidence to support the need for more attention in this age group.

According to the WHO, cancer is responsible for 365,000 deaths annually [22]. Because of that, the study tried to investigate the types of cancer. We observed that oesophageal cancer, lung cancer, and prostate cancer accounted for the highest proportion. In the general public, the general national cancer mortality demographic data show that among males, prostate cancer (25.1%) is the most common cause of mortality followed by oesophagus cancer (16.0%) and liver cancer at 8.2%, while in females, it is cervical cancer (24.2%), breast cancer (12.5%), oesophageal cancer (7.7%), and liver cancer (5.8%) [22]. Our findings differ slightly from the national figure with oesophageal cancer being the most common cause of mortality.

One of the limitations for this study is that some records did not provide sufficient demographic data. In addition, the antecedent causes of death were not confirmed due to paucity of postmortem reports. Finally, other outcomes that could impact the health systems such as hospital costs and readmission rate could not be explored.

## 6. Conclusion

Our study has highlighted the major health challenges that cause older adults to be hospitalized in a regional referral hospital population. In addition, the study has shown that there is poor prognosis in this age group and requires further comprehensive investigations.

## Figures and Tables

**Table 1:** Demographic characteristics of older adult patients at MRRH.

Characteristics	*n* (%), *n* = 813
Age median (IQR)	70 (65–80)

Gender	
Male	386 (47.5)
Female	427 (52.5)

Tribe	
Munyankole	517 (63.6)
Mukiga	43 (5.3)
Muganda	30 (3.7)
Others	9 (1.1)
Not indicated	214 (26.3)

Marital status	
Single	97 (11.9)
Married	352 (43.3)
Not indicated	364 (44.8)

Level of education	
None	283 (34.8)
Primary	22 (2.7)
Secondary	5 (0.6)
University/tertiary	25 (3.1)
Not indicated	478 (58.8)

Type of admission	
Referral	179 (22.0)
Self-referred	634 (78.0)

Admission ward	
Medical	355 (43.7)
Psychiatry	2 (0.3)
Obs and gyn	1 (0.1)
Surgical	84 (10.3)
Emergency	371 (45.6)

**Table 2:** Clinical conditions of older adult patients at admission in MRRH.

Disease condition	*n* (%)
Cancer	130 (14.5)
Stroke	94 (10.5)
Heart failure	76 (8.5)
Chronic obstructive pulmonary disease	56 (6.2)
Pneumonia	47 (5.2)
Head injury	45 (5.0)
Diabetes	43 (4.8)
Hypertension	36 (4.0)
Sepsis	35 (3.9)
Severe anemia	31 (3.4)
Severe malaria	25 (2.8)
Fractures	24 (2.7)
Intestinal obstruction	19 (2.1)
Renal disease	18 (2.0)
Hernia	13 (1.4)
Gastric outlet obstruction	12 (1.3)
Tuberculosis	11 (1.2)
Electrolyte imbalance	10 (1.1)
Upper gastrointestinal bleeding	10 (1.1)
Meningitis	9 (1.0)
Liver disease	9 (1.0)
Gangrene	8 (0.9)
Gastroenteritis	7 (0.7)
Dementia	5 (0.5)
Peritonitis	5 (0.5)
Alcohol intoxication	4 (0.4)
Oesophageal stricture	3 (0.3)
Others	104 (11.7)
Total	889 (100)

**Table 3:** Outcomes of older adult patients admitted at MRRH.

Outcome	*n* (%)
Patient was discharged	560 (68.9)
Patient referred for advanced care	18 (2.2)
Patient escaped from facility	38 (4.7)
Patient died	197 (24.2)
Total	813 (100)

**Table 4:** In-patient outcomes according to the ward of admission.

Outcome	Hospital wards					
	Medical	Psychiatry	Obs/gyn	Surgical	Emergency	Total
Discharged	276	2	0	40	242	560
Referred	10	0	0	1	7	18
Escaped	13	0	0	4	21	38
Died	56	0	1	39	101	197
Total	355	2	1	84	371	813

**Table 5:** Antecedent causes of death among older adult patients at MRRH.

Diagnosis made	Died, *n* (%)
Stroke	30 (15.2)
Cancer	21 (10.7)
Head injury	16 (8.1)
Heart failure	14 (7.1)
Sepsis	14 (7.1)
Renal disease	12 (6.1)
Pneumonia	9 (4.6)
Chronic obstructive pulmonary disease	8 (4.1)
Diabetes	7 (3.6)
Gastric outlet obstruction	5 (2.5)
Meningitis	5 (2.5)
Gangrene	4 (2.0)
Intestinal obstruction	4 (2.0)
Severe malaria	4 (2.0)
Alcohol intoxication	3 (1.5)
Fractures	3 (1.5)
Peritonitis	3 (1.5)
Dysentry	2 (1.0)
Gastroenteritis	2 (1.0)
Metabolic encephalopathy	2 (1.0)
Oesophageal stricture	2 (1.0)
Others	27 (13.7)
Total	197 (100)

## Data Availability

The data used to support the findings of this study are available from the corresponding author upon request.

## Abbreviations

MRRH: Mbarara Regional Referral Hospital
WHO: World Health Organization
UNESCO: United Nations Educational, Scientific, and Cultural Organization.
